# No healthy schools without healthy teachers: a scoping review on implementation determinants, strategies and outcomes of mental health-promoting interventions for school teachers

**DOI:** 10.1186/s12889-026-26589-w

**Published:** 2026-02-11

**Authors:** Katharina Sterr, Joachim Bachner, Daniel Alexander Scheller, Filip Mess, Simon Blaschke, Theres Mühlberg, Friederike Butscher, Jan Schmid-Ellinger

**Affiliations:** TUM School of Medicine and Health, Department of Health and Sport Sciences, Technical University of Munich, Am Olympiacampus 11, Munich, 80809 Germany

**Keywords:** School teachers, Mental health, Well-being, Intervention, Implementation, Workplace health promotion, School health promotion, CFIR, SISTER, IOF

## Abstract

**Background:**

The mental health and well-being of school teachers is critical not only for their individual health but also for the quality and stability of educational systems. Numerous interventions have been developed to address teachers’ mental health challenges, yet their implementation in everyday school settings remains limited. Understanding implementation determinants, strategies, and outcomes is essential for improving sustainable implementation, intervention effectiveness and broader public health impact. This scoping review explored how implementation is addressed in studies evaluating mental health-promoting interventions for teachers.

**Methods:**

Following Arksey and O’Malley’s (2005) and Levac et al.’s (2010) frameworks and PRISMA-ScR guidelines, we systematically searched Scopus and EBSCOhost up to April 2025. Studies were included if they evaluated an intervention targeting teachers’ mental health and reported at least one implementation aspect. Data extraction was guided by leading implementation science frameworks.

**Results:**

Of 4,062 identified records, 16 met the inclusion criteria. Most studies were primarily effectiveness-focused and assessed early-stage implementation rather than long-term implementation or sustainment. Implementation outcomes such as acceptability and feasibility were frequently reported but rarely grounded in implementation frameworks. Implementation determinants appeared in most studies, predominantly as post hoc barriers, with few studies assessing them a priori to guide implementation planning. Implementation strategies were commonly described but seldom explicitly labeled as such. Most studies examined implementation and intervention outcomes separately, limiting insights into how implementation processes influenced effectiveness. Nevertheless, several insights emerged, including the relevance of training and educating stakeholders, tailoring interventions to context, and strengthening relational dynamics, all examples of implementation strategies, as well as the importance of considering intervention content and implementation jointly.

**Conclusion:**

Although implementation determinants, strategies, and outcomes were reported in studies on teachers’ mental health interventions, reporting was often fragmented, unsystematic and rarely guided by established frameworks or terminology. Future research should adopt comprehensive, theory-informed approaches that link implementation and intervention content. From a public health perspective, aligning evidence-based interventions, addressing both organizational and individual levels, with context-sensitive implementation strategies is key to sustainably improving teachers’ mental health and strengthening schools as healthy, supportive environments.

**Supplementary Information:**

The online version contains supplementary material available at 10.1186/s12889-026-26589-w.

## Introduction

The well-being of teachers is central to the quality of the educational system. Yet, many school teachers suffer stress-related mental health issues, such as emotional exhaustion and burnout [1–3]. As a result, they often experience lower job satisfaction, higher intention to quit, poor sleep quality, and an overall decline in quality of life [4, 5]. Moreover, teachers’ mental health affects students, e.g. their motivation and academic achievement [6, 7], as it directly influences teaching quality [8]. Additionally, it is widely assumed that high levels of teacher stress lead to consequences beyond academic outcomes and negatively impact students’ mental health and well-being [9]. Poor mental health among teachers is also reflected in workforce challenges. In Germany, for example, high rates of both sick presentism and absenteeism have been reported, and three out of four teachers retire earlier than legally required [10–12]. This, in turn, intensifies the ongoing shortage of teaching staff [13].

The far-reaching consequences of poor teacher mental health underscore the need for targeted interventions – to improve individual well-being of teachers and thereby to support students’ mental health, academic success, and the overall stability of the educational system.

Given the importance of teachers’ mental health, it is not surprising that a substantial number of scientifically evaluated interventions address various facets of the topic, such as stress, signs of burnout, depression, and overall well-being, with varying degrees of intervention complexity [8, 9]. Meta-analyses indicate that interventions aiming at stress-reduction tend to yield the largest effects, while moderate effects have been observed for anxiety, depression, and well-being [14, 15]. Effects on burnout appear to range from small to moderate, possibly depending on the type of school at which teachers work [14, 15]. A closer examination of previous studies highlights certain intervention characteristics with particular potential. For example, blended interventions that combine web-based and face-to-face components seem especially effective in reducing burnout [16]. Mindfulness-, behavioral-, and cognitive-behavioral-based approaches have shown effectiveness in alleviating stress [9]. Further, recent research highlights the importance of workplace conditions within schools for teachers’ health and well-being [17]. Interventions that target both organizational-level factors, such as peer collaboration and supportive leadership, and individual-level factors, such as stress management, are considered particularly promising for promoting teachers’ mental health and well-being [18, 19].

However, Beames et al. conclude in their systematic review that “most of the programs examined required significant time, effort, and resources to deliver and complete. These programs may not translate well outside of research trials to real-world contexts due to teachers being time-poor” [15]. This quote highlights that often interventions that have been successfully tested for their effectiveness are not automatically implementable and sustainable in everyday school life [8, 9, 15, 20].

Overall, an imbalance can be observed in research on interventions to promote teacher mental health: while extensive analyses exist on the prevalence of mental health issues among teachers, the number of high-quality studies on the effectiveness of countermeasures is already lower and studies investigating implementation are even more scarce. Thus, while we theoretically know *what* works (intervention content), we lack knowledge on *how* these interventions must be implemented to be effective in real-world contexts. To address this gap, Lewis et al. therefore call for innovative research designs, and for synthesizing existing knowledge through reviews, in order to systematically build field-specific implementation-related evidence [21].

For such endeavors, implementation frameworks can provide orientation. They can guide theoretical thinking, the design of studies, and the interpretation of findings, while also offering a common terminology that supports a shared understanding of what constitutes implementation and what has been done during implementation efforts [22]. The Consolidated Framework for Implementation Research (CFIR) is one of the most widely used frameworks in implementation science [23–25] and groups key aspects of implementation, such as contextual influences (implementation determinants), actions taken to support the implementation process (implementation strategies), and indicators of implementation success (implementation outcomes) [26]. These aspects can be illustrated with a hypothetical school-based stress-reduction intervention.

- Implementation determinants are contextual factors that facilitate or hinder implementation [24]. For instance, one school may have space to create a relaxation room for teachers, while another has no appropriate staff room at all—conditions that strongly shape whether a stress-reduction intervention can be carried out during the school day and that probably differ from school to school.
- Implementation strategies are deliberate actions used to support implementation [27], such as providing teacher training or opportunities for peer exchange between schools on best practices.
- Implementation outcomes, such as acceptability and feasibility (early-stage), reflect whether teachers perceive an intervention as suitable and manageable in their everyday work. Without satisfactory levels of such outcomes, implementation and sustainment (late-stage implementation outcomes) is unlikely, thereby hampering effectiveness [26, 28].

Understanding these elements together is essential: teachers first need favorable conditions that allow them to implement an intervention (determinants), they need support that enables and empowers them to do so (strategies), and they must actually carry out the intervention as intended for a sufficient period of time (implementation outcomes) [25, 29, 30]. Only then can improvements in teachers’ mental health emerge, following a dose–response logic [31]. Put simply, interventions cannot be effective if they are not implemented.

However, despite the importance of implementation, little is known about how studies on teacher mental health interventions describe or assess these implementation aspects.

To address this gap, we decided to conduct a scoping review with the aim of mapping how existing studies on teacher mental health interventions report implementation determinants, strategies, and outcomes and explore to what extent studies assess them in relation to each other.

## Methods

To structure the review process, we adopted the first five stages of the methodological framework developed by Arksey and O’Malley and largely followed the extension by Levac et al. [32, 33], except for stage six (stakeholder consultation), which was not feasible within the scope of this study. Further, we ensured transparent reporting by following the PRISMA guidelines for scoping reviews [34].

### Stage 1: Identifying the research question

The first step in a scoping review is to define the research aim, as it informs the development of the search strategy and guides decisions about inclusion and exclusion criteria. In our case, the aim was to explore how implementation is addressed in studies evaluating mental health-promoting interventions for school teachers.

Based on Ashcraft et al.’s findings, we were aware that aspects of implementation might be inconsistently defined and operationalized in the literature [35]. Some studies may refer to implementation aspects without grounding their approach in established taxonomies or implementation frameworks, e.g. in terms of specifying the determinants, strategies or outcomes they assess [36]. To account for this variability, we used a broad and inclusive search strategy, which is detailed in the next stage.

### Stage 2: Identifying relevant studies

The search term included the following five components, each with terminological derivations or related terms: intervention (e.g. program), target population (teach*), setting (e.g. work), topic of intervention (e.g. mental*, well-being), implementation (e.g. implement*). Details of the search term are provided in Additional File A. To identify relevant studies, we systematically searched the databases Scopus and EBSCOhost in February 2024 for the initial search and in April 2025 for the update search, applying the same strategy to capture any newly published relevant studies. After deduplication, the initial search yielded 3638 and the update search 424, combined 4,062, English-language records. The search was restricted to non-review studies, and forward and backward citation tracking did not yield any additional records.

### Stage 3: Study selection

All records identified through the database searches were screened in a two-phase process using Rayyan, a web-based tool designed to facilitate blinded and collaborative screening [37]. In the first phase, titles and abstracts were independently screened by two reviewers out of eight members of the research team. Articles were excluded if they (a) did not report on an intervention, (b) focused solely on effectiveness evaluation without any mention of implementation aspects, (c) were not conducted with teachers in general education schools, or (d) did not address teacher well-being or mental health. Discrepancies between reviewers were resolved through discussion until consensus was reached.

**Fig. 1:**
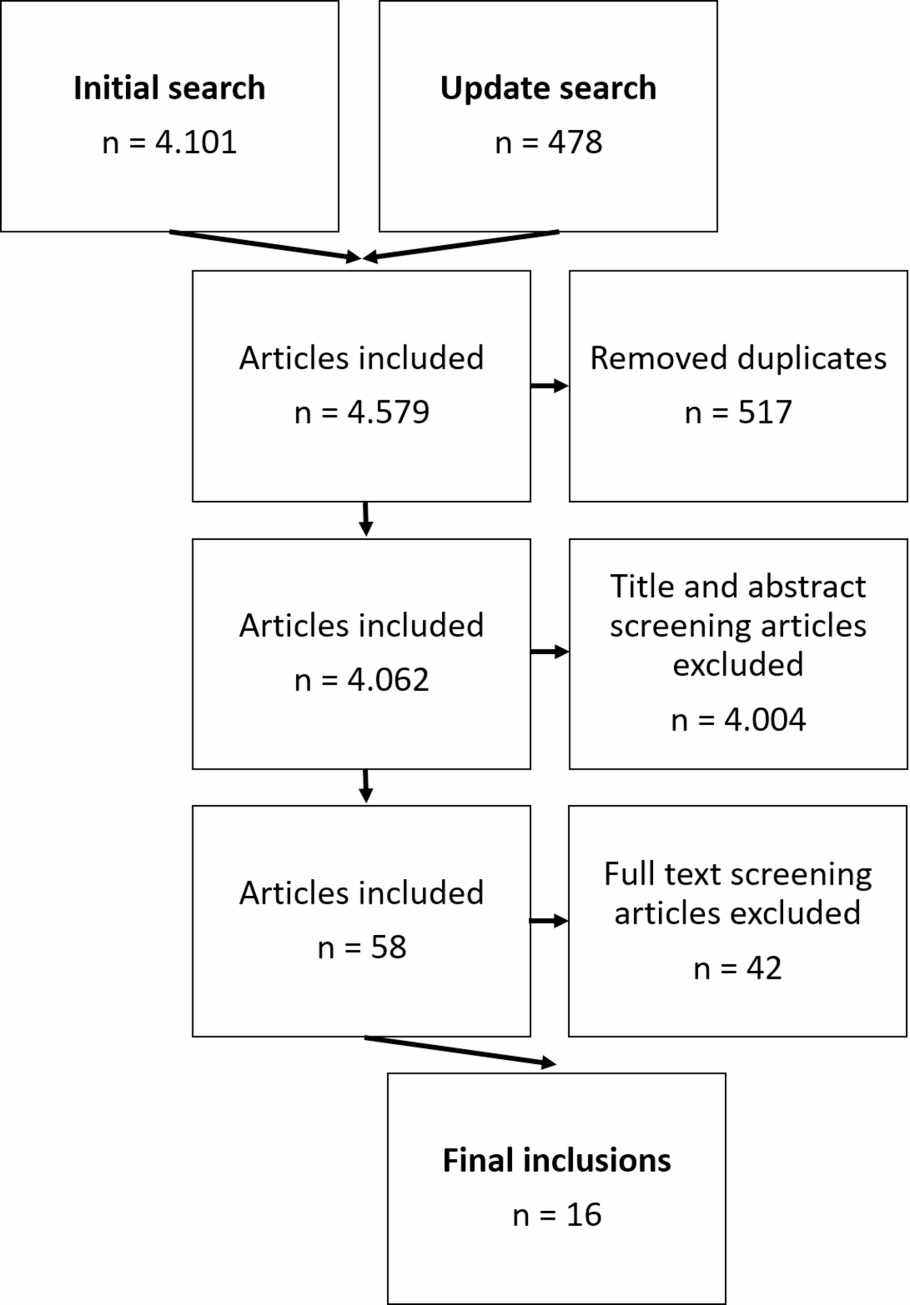
Flow diagram of the study selection process

This first screening phase resulted in 58 potentially relevant articles. In the second phase, again two reviewers independently assessed the full texts of these articles. After applying the same inclusion and exclusion criteria at this full-text level, 16 studies met all criteria and were included in the review. The full screening process is depicted in Fig. 1.

### Stage 4: Data extraction & charting

To systematically extract relevant information from the included studies, we used the updated CFIR codebook [26] for coding implementation determinants, the Implementation Outcomes Framework (IOF) [28] for coding implementation outcomes, and the School Implementation Strategies, Translating ERIC Resources (SISTER) taxonomy [38] for coding implementation strategies. Both the CFIR and the SISTER taxonomy consist of several domains or overarching categories, that group a set of determinants or strategies respectively. An overview of the structure of these frameworks, including domains and subcategories, is given in Table 1.

**Table 1:** Overview of applied implementation frameworks

Implementation Aspects	Framework/Taxonomy	Structure
Implementation determinants “CFIR implementation determinants capture setting-level barriers and facilitators that predict and/or explain *antecedent assessments* and/or *anticipated or actual implementation outcomes”.* (Damschroder et al., 2022, p. 8)[23, 26]	Consolidated Framework for Implementation Research (CFIR) (Damschroder et al., 2009, 2022)[23, 26]	5 domains - Innovation - Inner Setting - Outer Setting - Individuals - Process encompassing 48 constructs and 19 sub-constructs
Implementation strategies = “Implementation strategies are methods or techniques used to enhance the adoption, implementation, and sustainability of a [clinical] program or practice.” (Proctor et al., 2013, p. 2)[27]	School Implementation Strategies, Translating ERIC Resources (SISTER) (Cook et al., 2019)[38]	9 domains (overarching categories) - TES – Train and Educate Stakeholders - DSI – Develop Stakeholder Interrelationships - EIS – Use Evaluative and Iterative Strategies - AT – Adapt and Tailor to the Context - PIA – Provide Interactive Assistance - EC – Engage Consumers - CI – Change Infrastructure - UFS – Utilize Financial Strategies - SC – Support Clinicians (Practicioners) encompassing 75 strategies
Implementation outcomes = “Implementation outcomes are the effects of deliberate and purposive actions to implement new treatments, practices, and services.” (Proctor et al., 2011, p. 1)[28]	Implementation Outcomes Framework (IOF) (Proctor et al., 2011) [28]	8 implementation outcomes - Acceptability - Adoption - Appropriateness - Feasibility - Fidelity - Implementation Cost - Penetration - Sustainability

We developed an Excel-based data charting form (i.e. a structured extraction sheet for systematically organizing implementation-related information) informed by these guiding frameworks and refined it iteratively, as recommended by Levac et al. [33]. During the initial extractions, and as expected based on Ashcraft et al.’s findings, it became evident that uniformly coding implementation determinants, strategies, and outcomes across all studies was not feasible due to inconsistent and heterogeneous terminology [35]. We therefore adopted a two-step approach to support coding, charting, and transparent reporting. Examples detailing the coding and mapping process are provided in Table 2. First, we coded at the domain/category level for determinants (CFIR domains) and strategies (SISTER categories), rather than identifying explicit, fine-grained determinant or strategy terms. Second, if determinants, strategies, or outcomes were explicitly labeled as such and referenced a framework or taxonomy, we classified them as “explicitly named.” If they were labeled but without referencing a framework, we categorized them as “named.” Finally, if determinants, strategies, or outcomes were not explicitly labeled but embedded within the study description (e.g., methods, results, or discussion sections), we mapped them to the corresponding domains, categories or outcomes and labeled them as “attributed.” This structure enabled consistent mapping of implementation aspects across studies while allowing us to document variation in terminology and reporting clarity.

Data extraction was primarily conducted by the first author, who had the most experience in implementation research. To enhance rigor, resolve uncertainties, and ensure a shared interpretation, coded passages were regularly discussed within the research team.

**Table 2:** Examples of data extraction

Exemplary quotes	CFIR/SISTER/IOF	Coded as
Implementation strategies attributed to the SISTER taxonomy		
* “During step 1*,* at each school a working group was formed (consisted of the school principal and 2–3 employees) that was responsible for action planning (step 3) and implementation (step 4).”* (Bakhuys Roozeboom et al. 2024, Netherlands [47], p.189)	Develop Stakeholder Interrelationships Domain - Use advisory boards and workgroups - Build partnerships (i.e. coalitions) to support implementation - Identify and prepare champions	Develop Stakeholder Interrelationships (DSI)
* “Teachers […]*,* selected by the Senior Leadership Team*, *attended the one-day MHFA [Mental Health First Aid] for Schools and Colleges* *training.”* (Evans et al. 2022, UK [39], p. 924)	Train and Educate Stakeholders Domain - Conduct educational meetings	Train and Educate Stakeholders (TES)
* “Following discussions between the researchers and the LT [lead teacher] over 3 months*,* the PPI [positive psychology intervention] programmes were collaboratively designed by considering relevant theories*,* the existing school schedule*,* staff interests and availability.”* (Yeh & Barrington 2023, UK, [45], p. 3)	Adapt and Tailor to Context Domain - Promote adaptability - Tailor strategies	Adapt and Tailor to Context (AT)
Implementation determinants attributed to the CFIR		
* “Teachers reported ‘lack of time’ and ‘prioritising school-wide scheduled PD courses’ as the main reasons for not undertaking the teacher Decode PD.” (*Habgood et al. 2025, Australia, [50], p. 1544)	Inner Setting - Time constraints & relative priority	Inner Setting
“*We reported previously that the challenges encountered in implementation were due* *at least in part to support from senior leadership waning over time.” (*Kidger et al. 2021, UK, [41], p. 17)	Individuals Domain - High-Level Leaders’ support	Individuals Domain
* “The COVID-19 pandemic (started after T1) affected the ability of schools to give priority to the action plans.”* Bakhuys Roozeboom et al. 2024, Netherlands, [47], p. 193)	Outer Setting - Critical incidents	Outer Setting
Implementation outcomes attributed to the IOF		
* All teachers valued the online delivery of the program. They found the delivery method practical and interesting by declaring*,* ‘I could access the materials whenever I could and could study them over and over by practicing the skills when I faced certain problems.’* *(*Ghasemi et al. 2023, Iran, [49], p. 13)	Appropriateness: The “perceived fit, relevance, or compatibility of the innovation […] for a given practice setting, provider, or consumer; and/or perceived fit of the innovation to address a particular issue or problem.” Feasibility: The extent to which an innovation “can be successfully used or carried out within a given agency or setting.”	Appropriateness, Feasibility
* Over 65% of the participants consistently reported applying the content to both* *personal and professional contexts. (*Hepburn et al. 2021, Australia, [59], p. 17)	Adoption: The intention, initial decision, or action to try or employ an innovation.	Adoption
* One third of the teachers never received training […]. Another third had received limited training […]. Only one third received more than one year of training. Thus*,* although teachers evaluate the program positively*,* and perceive the school administration as supportive of the program*,* training is insufficient. This alone may hamper success of the intervention for teachers.* (Shechtman et al. 2001, Israel [53], p. 17)	Penetration: The integration of a practice within a service setting and its subsystems which can be calculated in terms of the number of providers who deliver a given service or treatment, divided by the total number of providers trained in or expected to deliver the service.	Penetration

### Stage 5: Collating, summarizing, and reporting the results

We summarized the charted data using descriptive statistics, including frequencies and distributions across study characteristics. In line with our research objectives, particular attention was paid to qualitatively identifying how studies address implementation determinants, strategies, and outcomes.

Further, we also aimed to identify which frameworks (if any) are used, and whether relations between implementation determinants, strategies, and outcomes as well as intervention outcomes are reported. These findings are presented narratively.

## Results

Table 3 provides an overview of the included studies, their designs, target populations and key characteristics of the interventions. Detailed information on extracted implementation aspects (determinants, strategies and outcomes) is presented in Table 4, which equals our data charting form.

**Table 3:** Overview of included studies: study design, target population, and key intervention characteristics

Authors, Year, Country	Study Design Type	School Setting and Target Population	Intervention Description	Level of Intervention	Intended Intervention (Mental Health) Outcome	Intervention Effectiveness
Bakhuys Roozeboom et al. 2024, Netherlands [47]	Quasi-experimental study	Primary schools (*N* = 30, 4 intervention, 26 control); Teaching and non-teaching staff (N not reported)	Name: Work Stress Prevention Approach Type: Organizational-level participatory intervention Duration: 3 years Delivery Mode: In-person, school-specific action plans; supported by risk assessments, logic model development, and monthly feedback	Organizational	Improve emotional exhaustion; reduce work stress determinants (e.g., leadership, job crafting, team culture)	Partly
Carbonero-Martín et al. 2022, Ecuador [42]	Non-randomized controlled trial	Primary schools (N not reported); Teachers (*N* = 351; 190 intervention, 161 control)	Name: Not specified Type: Emotional education program Duration: 8 weeks, 32 h Delivery mode: Online, asynchronous content, guided via manual	Individual	Improve emotional intelligence	Yes
Ebert et al. 2015, Germany [46]	Randomized controlled trial	Primary and secondary schools across Germany (N not reported); Teachers (*N* = 128)	Name: GET.ON Recovery Type: Internet-based self-help intervention Duration: 6 sessions over 6–8 weeks Delivery Mode: Online with email-based guidance	Individual	Improve psychological recovery, sleep quality, and well-being	Yes
Evans et al. 2022, UK [39]	Randomized controlled trial	Secondary schools (*N* = 25); Teachers and support staff (N not reported)	Name: WISE (Well-being in Secondary Education) Type: Mental Health First Aid training, Mental health awareness session, Peer support service Duration: 2 years Delivery Mode: In-person training sessions and in-school peer support implementation	Organizational	Improve mental health and well-being	Not assessed; implementation focus
Falck & Kilcoyne 1984, USA [58]	Pilot-Pre-post study	Schools (Type not specified, N not reported); School employees (teachers, nurses, counselors, principals) (N not reported)	Name: Not specified Type: Prevention-oriented health promotion and self-help program Duration: 7 weeks (5 sessions) training, 1 year implementation Delivery Mode: In-person, facilitator-led small group sessions using a flexible training manual	Organizational	Improve self-management, coping, physical fitness, nutrition, and overall well-being	Yes
Ghasemi et al. 2023, Iran [49]	Pilot-Randomized controlled trial	Schools (type not specified, N not reported); Teachers (*N* = 40)	Name: Not specified Type: Online Acceptance and Commitment Therapy (ACT) Duration: 3 sessions over 6 weeks Delivery Mode: Self-guided via Telegram with facilitator support	Individual	Improve psychological well-being	Yes
Habgood et al. 2025, Australia [50]	Pilot-Pre-post study	Secondary schools (*N* = 2); Teachers and students (Years 5–8; N not reported)	Name: Decode Mental Health and Wellbeing Program Type: Digital mental health literacy program Duration: 4 weeks Delivery Mode: Online; social media-style videos and content; teacher and student components	Individual	Improve mental health literacy, reduced stigma, increased help-seeking	Yes
Hepburn et al. 2021, Australia [59]	Pre-post study	Schools (type not specified, N not reported); Teachers (*N* = 24)	Name: Integrated Wellbeing and Stress Management Program Type: Combined mindfulness, yoga, education, and reflection Duration: 6 weeks Delivery Mode: In-person, after-school group sessions	Individual	Improve well-being; reduce stress	Yes
Jackman et al. 2025, USA [48]	Randomized controlled trial	Primary and secondary schools (N not reported); Teachers (*n* = 212 intervention, *n* = 202 control)	Name: Mindfulness for Humans (MfH) Type: Online self-directed mindfulness course Duration: 3 weeks, 15 sessions Delivery Mode: Online instructional videos and guided practices	Individual	Improve well-being	Yes
Kidger et al. 2016, UK [40]	Randomized controlled trial	Secondary schools (*N* = 6); Teachers and students (*N* = 509 teachers, *N* = 1060 students)	Name: WISE (Wellbeing in Secondary Education) Type: Mental Health First Aid (MHFA) training and peer support service Duration: 2-day MHFA training; peer support setup integrated into school year Delivery Mode: In-person training; in-school peer support	Organizational	Improve mental health and wellbeing (teachers and students)	Not assessed; implementation focus
Kidger et al. 2021, UK [41]	Randomized controlled trial	Secondary schools (*N* = 25); Teachers and students (*N* ≈ 1000 teachers, *N* ≈ 4000 students)	Name: WISE (Well-being in Secondary Education) Type: Mental Health First Aid training, Mental health awareness session, Peer support service Duration: 2 years Delivery Mode: In-person training sessions and in-school peer support implementation	Organizational	Improve mental health and well-being (teachers and students)	No
Mendelson et al. 2023, USA [54]	Feasibility/Pre-post study	Primary and secondary schools (*N* = 6); Teachers (*N* = 60)	Name: DeStress Monday at School Type: Online mindfulness intervention Duration: 9 weeks Delivery mode: Weekly online practices (email reminders + website)	Individual	Improve well-being, sleep and mental health (anxiety, depression); reduce stress	Yes
Oliveira et al. 2022, Portugal [51]	Pilot Quasi-experimantal study	Primary school in Brazil (*N* = 1); Teachers (*N* = 7)	Name: A+ Type: Online Social and Emotional Learning (SEL) intervention Duration: 10 weeks (50 h) Delivery mode: Blended online format; instructor-led synchronous sessions and asynchronous homework	Individual	Improve well-being and emotion regulation; reduce stress	Yes
Shechtman et al. 2001, Israel [53]	Non-randomized controlled trial	Primary schools (*N* = 97); Teachers (N not reported)	Name: Life Skills Training (LST) Program Type: Psychoeducational group intervention focusing on personal and interpersonal competencies Duration: 1 or 2 years of monthly 3-hour sessions Delivery Mode: Group-based training provided by school counselors	Individual	Improve well-being, perception of work climate and self-efficacy	Yes
Toropova et al. 2022, Sweden [44]	Randomized controlled trial	Primary schools (*N* = 19); School personnel (teachers, admin, pedagogues; *N* = 400)	Name: Guideline for prevention of mental ill-health Type: Organizational guideline implementation Duration: 12 months Delivery mode: Multifaceted (educational meeting, workshops, implementation teams, PDSA cycles) vs. single (educational meeting only)	Organizational	Improve adherence to guideline recommendations for preventing mental ill-health among school personnel	Not assessed; implementation focus
Yeh & Barrington 2023, UK [45]	Case Study	Primary school (*N* = 1); Teachers (*N* = 54)	Name: Tailored Positive Psychology Interventions (PPIs) Type: Whole-school and individual intervention Duration: 7–8 weeks Delivery mode: Brief staff-meeting sessions and in-depth self-interest group course	Organizational	Improve well-being (emotional, relational, work-related), work-life balance	Yes

**Table 4:** Implementation aspects extracted from included studies

Authors, Year, Country	Approach to Implementation Evaluation	Application of an implementation framework/model/ theory	Implementation Determinants (a priori/post hoc/both)	Implementation Strategies	Implementation Outcomes	Aspects Linked
Bakhuys Roozeboom et al. 2024, Netherlands [47]	Quantitative	Yes; Intervention Process Measure (IPM) (Randall et al. 2009)	Both; A priori barriers: time constraints, workload (Inner Setting). Post hoc barriers: low priority due to COVID-19 (Outer Setting). Facilitators: leadership commitment, staff involvement (Individuals).	Attributed; Delivering MHFA training (TES), Establish implementation and peer support teams (DSI), Tailoring to school context (AT)	Attributed; Acceptability (high in engaged schools), Feasibility (varied), Fidelity (inconsistent), Appropriateness (perceived useful)	Yes; formally assessed (implementation process and outcomes statistically linked to MH outcomes)
Carbonero-Martín et al. 2022, Ecuador [42]	Interpretative	No	Not assessed	Explicitly named; group segmentation (AT); Attributed; Delivering psychoeducational training (TES), Adapting intervention to teacher context (AT)	Not reported	No; not assessed
Ebert et al. 2015, Germany [46]	Quantitative	No	Post hoc only; Barriers: workload, time constraints (Inner Setting)	Attributed; Tailored digital delivery (AT), Facilitator support via email (PIA), Guided content via modules (TES)	Named; Adherence (moderate to high), Satisfaction (positive)	Yes; formally assessed and interpreted (usage and adherence linked to effectiveness)
Evans et al. 2022, UK [39]	Qualitative	Yes; Acceptability of healthcare interventions (Sekhon et al. 2017)	Post hoc only; Barriers: confidentiality concerns, complex needs (Individuals), culture, workload, accountability (Inner Setting)	Attributed; Staff awareness sessions (TES), MHFA training (TES), Establishing peer teams (DSI)	Explicitly named; Acceptability (initially high, then low)	Yes; interpreted (low acceptability linked to low uptake linked to ineffectiveness)
Falck & Kilcoyne 1984, USA [58]	Mixed methods	No	Post hoc only; Facilitators: program facilitators and administrative support (Innovation); Both barriers & facilitators in different schools: leadership support & resources (Inner Setting)	Attributed; Training school-based facilitators (TES), Peer-to-peer delivery (DSI), continuous support (PIA)	Named; Feasibility (moderate), Attributed; Fidelity (inconsistent)	Yes; interpreted (some discussion of implementation quality influencing effectiveness)
Ghasemi et al. 2023, Iran [49]	Mixed methods	No	Post hoc only; Barriers: lack of formal support (Innovation), resource constraints (Inner Setting). Facilitators: motivation (Individuals)	Attributed; Digital program delivery (AT), Minimal facilitator support (PIA)	Attributed; Acceptability (positive), Feasibility (reported), Appropriateness (fit reported)	Yes; interpreted (training uptake discussed in relation to post-intervention outcomes)
Habgood et al. 2025, Australia [50]	Mixed methods	Yes; AIM, IAM and FIM (Weiner et al. 2017)	Post hoc only; Barriers: time constraints, relative priority (Inner Setting), technical difficulties, resistance to change, uneven engagement (Individuals).	Attributed; Staff co-design of intervention (AT), Flexibility of delivery (AT), Use of relatable content (TES)	Explicitly named; Feasibility, Acceptability, Appropriateness (moderate to high); Implementation (varied)	Yes; interpreted (engagement levels interpreted as influencing perceived impact)
Hepburn et al. 2021, Australia [59]	Qualitative	No	Post hoc only; Barriers: time constraints (Inner Setting), lack of clarity on expectations (Innovation)	Attributed; Experiential workshops and group discussions (TES), Peer sharing (DSI)	Attributed; Acceptability (high), Feasibility (challenged by time), Adoption (moderate)	No; not assessed
Jackman et al. 2025, USA [48]	Mixed methods	No	Post hoc only; Barriers: time constraints, workload (Inner Setting)	Attributed; Self-guided digital content (TES), Minimal prompting (PIA)	Named; Adherence, Acceptability (positive), Feasibility (high)	Yes; formally assessed and interpreted (adherence and intervention effectiveness)
Kidger et al. 2016, UK [40]	Mixed methods	No	Post hoc only; Barriers: low awareness, confidentiality concerns (Individuals)	Attributed; MHFA and peer support training (TES), Peer support infrastructure (DSI), Awareness sessions (TES)	Named; Acceptability (positive), Feasibility (partial), Appropriateness (mixed); Attributed; Adoption (partial)	Yes; interpreted (barriers and potential future implementation outcomes)
Kidger et al. 2021, UK [41]	Quantitative	No	Post hoc only; Barriers: lowered leadership support (Individuals)	Attributed; MHFA and peer support training (TES), Implementation manual (PIA), Staff awareness session (TES)	Not reported	Yes; interpreted (potentially low implementation led to lack of effectiveness)
Mendelson et al. 2023, USA [54]	Mixed methods	Yes; Feasibility (Bowen et al. 2009)	Both; A priori barriers: time constraints (Inner Setting), student engagement (Individuals). Post hoc barriers: time constraints (Inner Setting), student engagement (Individuals), interface issues (Innovation); Facilitators: flexibility (Innovation), teacher buy-in (Individuals)	Attributed; Weekly digital practices (AT), Teacher feedback integration (UEIS), Self-guided use (TES)	Named; Acceptability (high), Demand (moderate), Implementation (partial) as dimensions of feasibility	Yes; formally assessed (usage statistically linked to MH outcomes)
Oliveira et al. 2022, Portugal [51]	Quantitative	Yes; Program Implementation Model (Berkel et al. 2011)	Both; A priori barriers and facilitators in different study; Post hoc barriers: pandemic disruption (Outer Setting), time constraints (Inner Setting).	Attributed; Group-based compassion training (TES), In-person sessions (PIA)	Explicitly named; Implementation Quality; Group Responsiveness Attributed; Acceptability (high)	Yes; formally assessed and interpreted (higher group responsiveness and perceived greater MH outcome)
Shechtman et al. 2001, Israel [53]	Quantitative	No	Post hoc only; Facilitators: support materials (Innovation).	Attributed; Counselor-led sessions (PIA), School resource distribution (CI), Training sessions (TES)	Attributed; Fidelity (low), Acceptability (positive)	Yes; formally assessed (several process variables - implementation determinants and strategies (training! ) linked to outcomes)
Toropova et al. 2022, Sweden [44]	Quantitative	Yes; COM-B Model (Michie et al. 2011)	Both; A priori barriers: knowledge (Individuals), peer and organizational support (Inner Setting), role clarity (Individuals), time constraints (Inner Setting), motivational aspects (Individuals). Post hoc barriers: organizational change (Inner Setting), lack of stakeholder support (Inner Setting).	Explicitly named; Educational meetings (TES), Implementation teams (DSI), PDSA cycles (UEIS)	Named; Adherence (moderate)	Yes; formally assessed (implementation strategies matched to COM-B determinants; adherence measured)
Yeh & Barrington 2023, UK [45]	Qualitative	No	Post hoc only; Barriers: resistance due to negative experiences (Individuals), time constraints (Inner Setting).	Attributed; Teacher co-design of PPIs (AT), School leader support and engagement (DSI), Embedded short practices in routine (AT), Informal peer-led sustainability (DSI)	Named; Acceptability (high for some, mixed for others), Feasibility (moderate), Adoption (partial), Appropriateness (strong), Fidelity (varied)	Yes; interpreted (teacher experiences and implementation factors discussed in relation to impact)

### Characteristics of included studies and interventions

The included studies (*N* = 16) were published between 1984 and 2025, with most studies (12/16) being published after 2020. Four studies were conducted in the UK, three studies in the US and two in Australia. The remaining seven studies took place in the Netherlands, Germany, Sweden, Portugal, Ecuador, Iran and Israel. Seven studies equally addressed aspects of effectiveness and implementation, five studies focused on effectiveness and reported process/implementation data on the side, and four studies were process/implementation evaluation studies only. Study designs ranged from randomized controlled trials (7/16), non-randomized controlled trials (2/16), quasi-experimental designs (2/16) to simple pre-post designs (4/16) and one case study. Five of the 16 studies were conducted as feasibility or pilot studies. The studies addressed a broad range of mental-health-related outcomes (see Table 3), with well-being (12/16) being the most common. Most of the interventions targeted teacher mental health at the individual level (9/16), focusing on strengthening personal coping strategies and resources through behavioral prevention approaches (e.g., mindfulness training, stress management). In contrast, only five interventions addressed organizational-level factors by implementing structural or procedural changes within the school setting, with three of these studies being conducted within the framework of the same intervention (WISE intervention; [39–41]). For the evaluation of the implementation aspects, six studies each used a mixed-method approach and a quantitative approach, three studies applied a qualitative approach, and one study discussed implementation without any empirical data reported [42]. 

### Implementation aspects

Of the included 16 studies, ten studies did not use any implementation framework or model to ground their design and evaluation, five studies applied some kind of implementation framework or model to at least one part of their implementation design or evaluation and only one study fully applied a model (COM-B model by Michie et al. [43]) to their study planning and evaluation [44].

#### Implementation determinants

Almost all studies did, in some way, report implementation determinants (15/16), with 11 studies reporting barriers post intervention, and four studies reporting both a-priori and post-hoc determinants. Overall, 18 different implementation determinants were identified. Of the reported determinants, most were coded in the *Individuals Domain* (8/18), followed by the *Inner Setting Domain* (4/18), *the Innovation Domain* (4/18) and least the *Outer Setting Domain* (2/18). The most reported barriers were time constraints (10 times; *Inner Setting*) and workload (5 times; *Inner Setting*). Both teacher/staff engagement and leadership support (both *Individuals Domain*) appeared as both a frequent barrier (if lacking; 6 times) and a facilitator (if present; 8 times).

#### Implementation strategies

Regarding implementation strategies, most studies did not name them as such (14/16), except for Carbonero-Martín et al. [42] and Toropova et al. [44]. However, all studies reported some kind of implementation strategy. The mapping of the identified strategies to the SISTER domains [38] resulted in the following distribution: *Train and Educate Stakeholders* (13/16), *Adapt and Tailor to Context* (8/16), *Develop Stakeholder Interrelationships* (6/16), *Provide Interactive Feedback* (6/16), *Use Evaluative and Iterative Strategies* (2/16), and *Change Infrastructure* (1/16).

#### Implementation outcomes

Implementation outcomes were reported by almost all studies (14/16) with seven studies using some kind of term commonly used in implementation and/or process evaluation (9/14). The most reported and/or attributed implementation outcome was *acceptability* (11/14), followed by *feasibility* (8/14), *appropriateness* (5/14), *fidelity* (4/14), and *adoption* (3/14) and *adherence* (3/14).

### Linkage of implementation aspects and intervention

Regarding linking implementation aspects (determinants, strategies, outcomes) between themselves (4/16), or implementation aspects and intervention outcomes (14/16), most studies did at least partly interpret implementation aspects and intervention together, with seven studies formally assessing such pathways, mostly between implementation outcomes (e.g. adherence) and intervention effectiveness.

## Discussion

The aim of this study was to identify which implementation determinants, strategies, and outcomes were addressed in the existing literature on teacher mental health interventions and to explore the extent to which studies assessed the relations between these implementation aspects. To ensure conceptual clarity throughout the scoping review, we applied the CFIR framework for determinants, the SISTER taxonomy for strategies and the IOF for outcomes [24, 28, 38].

An initial insight concerned the nature of the included studies. As reported in the Results, only four studies were dedicated implementation or process evaluations, seven addressed both implementation/process and effectiveness equally and five were primarily effectiveness studies that reported some implementation-related information on the side. This heterogeneity in study purposes already hints to a limitation in depth and specificity with which implementation could be expected to be addressed. Further, this variation in study focus was reflected in inconsistent terminology and often vague conceptualizations of implementation. Most studies did not report using any implementation theory, model, or framework to guide their planning or evaluation (10/16). As a result, coding, mapping, and comparing implementation aspects across studies was challenging. However, our structured charting approach (e.g., domain-level coding and category labels) helped us approximate a consistent interpretation across heterogeneous reporting styles. In the following sections, we discuss our findings from an implementation research and practice perspective.

### Implementation aspects

#### Implementation determinants

Implementation determinants reflect contextual factors that either facilitate or hinder implementation [24]. In this review, most studies reported implementation factors post hoc, with few collecting a priori information on anticipated barriers or facilitators. Most post hoc determinants fell within the *Inner Setting*, highlighting school-internal challenges such as time constraints [45, 46], workload [47, 48], and limited resources [49]. Additional determinants fell within the *Individuals Domain*, including teachers’ concerns about confidentiality and stigma [39, 40], resistance to change [45, 50], and varying levels of leadership support [39, 47]. Leadership support was described as a facilitator when present [47] and as a barrier when absent [39]. Further determinants related to the *Innovation Domain*, such as a lack of technical support from deliverers [48, 49], and the *Outer Setting*, such as disruptions due to the pandemic [47, 51].

Overall, a wide range of implementation determinants was identified, with some determinants being reported consistently across studies (e.g., time constraints), while others varied substantially between school contexts (e.g., leadership support). While certain factors appear to shape implementation contexts consistently, indicating systemic conditions, the findings also underscore that implementation challenges differ across school settings, aligning with existing research on school-based implementation [52]. This highlights the importance of conducting a priori assessment of determinants to guide context-sensitive tailoring of strategies [30]. In other words, implementation is anticipated to work best when teachers’ and schools’ specific needs are understood in advance, including systemic conditions such as time constraints, and strategies are tailored accordingly [30].

#### Implementation strategies

Regarding implementation strategies, only two studies [42, 44] explicitly labeled their implementation strategies as such, however, nearly all included studies described activities that served an implementation-supporting function. Using the SISTER taxonomy allowed us to map these activities systematically across studies. The most frequently identified strategy category was *Train and Educate Stakeholders*, which typically targeted teachers as the primary implementers of interventions. This aligns with findings by Ashcraft et al., who reported strategies from this category to be the most commonly used strategies in school-based health interventions [35].

*Train and Educate Stakeholders* strategies took various forms across the included studies. Shechtman et al. investigated different training modalities and found that substantial training duration, two years instead of one, was necessary for teachers to feel confident enough to implement the intervention as intended [53]. Similarly, Toropova et al. used educational meetings, illustrating its centrality in preparing teachers for guideline-adherent mental health support practices [44]. Importantly, expert consensus work also highlights *Train and Educate Stakeholders* strategies as both feasible and highly relevant for school-based implementation [30], further underscoring their relevance across studies.

A second prominent strategy domain was *Adapt and Tailor to Context*, which was evident across interventions implemented at both the individual and organizational level [45, 49, 50, 54]. Tailoring involved providing flexibility in which, how, and when intervention components were implemented, allowing for better alignment with local routines, scheduling constraints or personal preferences. This finding is consistent with the growing literature on modular intervention design, which suggests that offering adaptable components (e.g., optional training units or adjustable delivery formats) facilitates contextual fit and may improve implementation *fidelity* and *acceptability* [55–57].

A third commonly mapped strategy domain was *Develop Stakeholder Interrelationships*. Several studies highlighted the importance of relational processes for successful implementation. Falck and Kilcoyne exemplified this by combining *Train and Educate Stakeholders*, *Adapt and Tailor to Context*, and *Develop Stakeholder Interrelationships* strategies in a peer-to-peer facilitation model, training teachers to act as implementation facilitators and supporters for their colleagues [58]. This model positioned teachers as both recipients and agents of implementation, illustrating an approach that blends capacity-building with relational support. In the WISE intervention [39], peer support or supervision was also embedded within the intervention content itself, further blurring the line between intervention and implementation. While peer-based approaches were generally seen as acceptable, concerns about confidentiality and stigmatization hindered their consistent use [39]. In contrast, a structured peer-debriefing format as seen in Hepburn et al.’s mindfulness program was experienced as reinforcing and motivating [59].

Taken together, these findings demonstrate that teacher mental health interventions rely on strategies that (1) build implementer capability (*Train and Educate Stakeholders*), (2) allow for contextual fit (*Adapt and Tailor to Context*), and (3) strengthen relational dynamics within school teams (*Develop Stakeholder Interrelationships*). However, their successful application appears highly context-dependent, shaped by constraints and facilitators at both the individual and the organizational level.

#### Implementation outcomes

Regarding implementation outcomes, only three studies used an implementation outcomes framework, which is consistent with findings from a recent review on experimentally tested implementation outcomes [35]. Most studies reported early-stage outcomes such as *acceptability* and *feasibility* only, used participation rates as a proxy for implementation outcomes, or inferred outcomes indirectly from anecdotal reports. This pattern again reflects the types of studies included in this review: 12/16 were not explicitly focusing on implementation evaluation and reported such aspects only alongside effectiveness findings. Current literature emphasizes the importance of assessing clearly defined and observable implementation outcomes, such as *adoption*, *penetration*, or *sustainment*, to build cumulative knowledge on what works in real-world school settings over time [29]. Relying primarily on teachers’ perceived *acceptability* or *feasibility* limits interpretability, as these perceptions do not necessarily translate into actual implementation behavior [29]. Both intervention content and implementation strategies may need to be adapted when observable outcomes indicate insufficient implementation or sustainment.

The WISE intervention [39–41] provides an illustrative example of why early *acceptability* alone is insufficient. Although WISE was perceived as highly acceptable and feasible in the pilot study [40], teachers in the main study reported that the intervention did not address deeper system-level factors affecting their mental health [39, 41]. As a result, *acceptability* declined, and implementation levels remained low [39]. This example highlights that implementation outcomes must be interpreted in relation to contextual determinants and intervention content and cannot be understood without considering these upstream factors.

Together, these findings underscore the need for more systematic and integrated assessment of implementation outcomes in teacher mental health research. They also set the stage for examining whether, and how, the included studies linked implementation aspects and intervention effectiveness.

### Linkage of implementation aspects and intervention

Overall, most studies reported implementation determinants, strategies, implementation outcomes, and intervention effectiveness in parallel rather than connecting them in an integrated manner. Yet such linkage is essential for understanding whether specific strategies address identified barriers, how they influence implementation outcomes, and under which contextual conditions they translate into effectiveness gains [29].

A small number of studies, however, provided more explicit insights into these processes.

Toropova et al. [44] offered the clearest example: as the only study in our sample that experimentally tested implementation strategies, they compared a single *Train and Educate Stakeholder* strategy (an educational meeting) with a multicomponent strategy bundle. Interestingly, they found no added benefit of the bundle over the single strategy regarding implementation success (guideline adherence), demonstrating that “more strategies” do not necessarily lead to better outcomes. Instead, they argued that strategies must be selected based on an understanding of context-specific determinants and planned collaboratively with stakeholders to achieve the best possible match [44]. Their study also illustrates a broader methodological challenge: full-scale experimental testing of implementation strategies is often infeasible in applied school settings due to resource constraints [60]. Alternative approaches that balance rigor and feasibility, including configurational methods, might be useful [25].

A second example, building on the WISE intervention discussed above, illustrates why integrating intervention content with implementation determinants, strategies and outcomes is essential. While WISE demonstrated high early-stage *acceptability* in the pilot study, its limited attention to system-level organizational root causes of poor teacher mental health hindered uptake in the main trial [39, 40]. The study by Bakhuys-Roozeboom et al. provides a complementary perspective. Their organizational-level intervention successfully improved several system-level determinants, most notably leadership quality, yet did not produce immediate improvements in emotional exhaustion [47]. Taken together, these findings illustrate that addressing system-level determinants is essential for creating the conditions under which implementation and mental health improvements can occur, but that effects on mental health may require longer timeframes to emerge.

Across studies, this interdependence of intervention content, contextual determinants, selected strategies, observable implementation outcomes and intervention effectiveness was visible but rarely made explicit. Strengthening this linkage is critical for advancing understanding of how teacher mental health interventions work in practice, under which conditions they succeed, and why implementation (or lack of it) mediates their effectiveness.

Ultimately, the goal of implementing mental health interventions is to sustainably improve teachers’ mental health and well-being. Achieving this requires attention to both *what* is being delivered (the content of the intervention) and *how* it is delivered (the implementation). An evidence-based intervention alone will not lead to meaningful change if it is not implemented in practice. Conversely, even a flawlessly executed implementation process will have limited impact if the intervention does not address the root causes of teachers’ mental health challenges. Future efforts must therefore adopt a dual focus, ensuring alignment between intervention content, implementation strategies, and the complex realities of school settings [61, 62].

Beyond implementation-specific implications, these findings also reinforce the broader public health relevance of supporting teachers’ mental health. Teachers’ well-being shapes teaching quality, student learning, and the overall resilience of the educational system [6, 8, 9]. In line with the principle of “no healthy schools without healthy teachers,” sustainable improvements require interventions that integrate organizational, system-level and individual-level approaches with context-sensitive implementation strategies, enabling schools to function as healthy and supportive environments for all members of the school community [55].

### Challenges and limitations

This review faced challenges and has some limitations that should be considered when interpreting the findings. First, mapping the implementation-related information reported in the included studies onto established implementation terminology was difficult due to heterogeneous and often inconsistent reporting. Because many studies did not explicitly label determinants, strategies, or outcomes, interpretive coding was required. However, following an iterative and reflexive charting approach, as recommended by Levac et al., allowed us to adapt the extraction framework throughout the process and more accurately capture how implementation was described [33].

Second, data extraction was conducted primarily by one author. According to the JBI Manual for Evidence Synthesis, when double extraction is not feasible, alternative approaches such as team-based discussion are appropriate, provided methods are transparently reported [63]. Consistent with this guidance, coded passages and mapping decisions were regularly reviewed within the research team in a reflexive process, helping to ensure shared interpretation and mitigate subjective bias.

Despite these challenges, the use of established implementation frameworks and iterative extraction process enabled a structured synthesis of the available evidence and identification of critical gaps that inform directions for future research.

### Implications for future research

The findings of this review point to several implications for strengthening research and practice in teacher mental health promotion. First, future studies should systematically apply implementation frameworks to guide the design, delivery and evaluation of interventions, and use consistent terminology in describing implementation determinants, strategies, and outcomes. Doing so would facilitate clearer reporting and enhance comparability across studies [22].

Second, there is a need for more comprehensive, theory-informed implementation evaluations that go beyond reporting single, early-stage indicators such as *acceptability* or *feasibility*. Future research should assess implementation aspects in a linked manner and make use of observable implementation outcomes (e.g., *adoption*,* penetration*,* sustainment*) to better understand how interventions function in real-world school settings over time [29]. Given the challenges of conducting full-scale experimental testing of implementation aspects in applied school settings, alternative methodological approaches are needed. Comparative configurational methods, such as coincidence analysis, offer promising potential [21, 25, 64]. When high-quality process data are available, these mathematically grounded, set-theoretic methods can identify context-specific combinations of determinants, strategies and intervention components that jointly contribute to successful implementation and intervention outcomes, providing a resource-sensitive way to advance causal understanding without requiring extensive experimental infrastructure [25, 64].

Third, future research should place greater emphasis on long-term implementation and sustainment studies, as many existing studies focus on early-stage or pilot evaluations. Understanding how interventions are adopted, adapted and sustained over time, particularly within diverse school contexts, remains an important evidence gap.

Fourth, on a practical level, the implementation strategy domains of *Train and Educate Stakeholders* and *Developing Stakeholder Interrelationships* emerged as especially relevant in school settings. However, these strategies should be selected and adapted based on context-specific determinants (*Adapt and Tailor to Context*), ideally in collaboration with school staff, including teachers as both the target group and key implementers.

Finally, intervention content and implementation processes are interdependent. Efforts to promote teacher mental health must therefore align both *what* is delivered and *how* it is delivered with the structural, organizational and relational realities of school environments to achieve sustainable improvements in teacher well-being.

## Conclusion

Overall, this scoping review of research on mental health-promoting interventions for school teachers identified specific implementation challenges: most included studies focused more on effectiveness than on implementation aspects, relied on early-stage implementation indicators and rarely applied implementation frameworks and established terminology. Consequently, implementation was often described in fragmented and inconsistent ways. These findings underscore the need for comprehensive, theory-informed implementation evaluation to capture the interdependence of implementation determinants, strategies, outcomes and intervention content and to advance both future research and practice. Sustainable improvements in teachers’ mental health will depend on aligning evidence-based interventions, addressing both organizational and individual levels, with context-sensitive implementation strategies. From a public health perspective, and in line with the principle of *no healthy schools without healthy teachers*, such alignment is essential to strengthen schools as environments that promote health, learning and well-being for the entire school community.

## Supplementary Information

Supplementary Material 1.

## Data Availability

All data generated through analysis of the included studies are included in this published article and its supplementary information files.

## Abbreviations

CFIR: Consolidated Framework for Implementation Research
SISTER: School Implementation Strategies, Translating ERIC Resources
IOF: Implementation Outcomes Framework
TES: Train and Educate Stakeholders (used in Table 4)
AT: Adapt and Tailor to Context (used in Table 4)
DSI: Develop Stakeholder Interrelationships (used in Table 4)
PIA: Provide Interactive Feedback (used in Table 4)
UEIS: Use Evaluative and Iterative Strategies (used in Table 4)
CI: Change Infrastructure (used in Table 4)
MH: Mental Health (only used in Tables 3 and 4)
MHFA: Mental Health First Aid (only used in Tables 3 and 4)
