# Supplementary material for: No healthy schools without healthy teachers: a scoping review on implementation determinants, strategies and outcomes of mental health-promoting interventions for school teachers
Source: BMC Public Health. 2026 Feb 11;26:710. doi: 10.1186/s12889-026-26589-w (PMC12931062; doi:10.1186/s12889-026-26589-w)
Supplement: Supplementary file 1 — Supplementary Material 1. [file 12889_2026_26589_MOESM1_ESM.docx]

|  |  | OR 🡪 | | | | | |
| --- | --- | --- | --- | --- | --- | --- | --- |
| **Intervention** | AND 🡪 | interv* | program* | train* |  |  |  |
| **Target population** |  | teach* |  |  |  |  |  |
| **Setting** |  | work | occupation* |  |  |  |  |
| **Topic of intervention** |  | mental* | psych* |  |  |  |  |
| **Topic of intervention** |  | health* | wellbeing | well-being | „well being“ | „quality of life“ |  |
| **Implementation** |  | adopt* | implement* | maint* | sustain* | strateg* | process |

((TITLE-ABS-KEY(interv*) OR TITLE-ABS-KEY(program*) OR TITLE-ABS-KEY(train*))

AND

(TITLE-ABS-KEY(teach*))

AND

(TITLE-ABS-KEY(work) OR TITLE-ABS-KEY(occupation*))

AND

(TITLE-ABS-KEY(health*) OR TITLE-ABS-KEY(wellbeing) OR TITLE-ABS-KEY(well-being) OR TITLE-ABS-KEY(“well being”) OR TITLE-ABS-KEY(“quality of life”))

AND

(TITLE-ABS-KEY(mental*) OR TITLE-ABS-KEY(psych*))

AND

(TITLE-ABS-KEY(strateg*) OR TITLE-ABS-KEY(process) OR TITLE-ABS-KEY(adopt*) OR TITLE-ABS-KEY(implement*) OR TITLE-ABS-KEY(Maint* ) OR TITLE-ABS-KEY(Sustain*)))
